# Long-Term Outcomes of Allogeneic Ocular Surface Reconstruction: Keratolimbal Allograft (KLAL) Followed by Penetrating Keratoplasty (PK)

**DOI:** 10.1155/2020/5189179

**Published:** 2020-04-14

**Authors:** Katarzyna Krysik, Dariusz Dobrowolski, Dorota Tarnawska, Edward Wylegala, Anita Lyssek-Boroń

**Affiliations:** ^1^Department of Ophthalmology with Pediatric Unit, St Barbara 5th Regional Hospital, Trauma Centre, Medykow Square 1, 41200 Sosnowiec, Poland; ^2^Chair and Clinical Department of Ophthalmology, School of Medicine with the Division of Dentistry in Zabrze, Medical University of Silesia, Panewnicka 65 Str., 40760 Katowice, Poland; ^3^Department of Ophthalmology, District Railway Hospital, Panewnicka 65, 40-760 Katowice, Poland; ^4^Department of Biophysics and Molecular Physics, A. Chelkowski Institute of Physics, Silesian Center for Education and Interdisciplinary Research, University of Silesia, 41-500 Chorzów, 75 Pułku Piechoty 1A, Poland

## Abstract

**Purpose:**

Long-term results of the patients with total LSCD, who had undergone keratolimbal allograft (KLAL) for limbal reconstruction followed by penetrating keratoplasty (PK).

**Methods:**

The study analyzes surgical treatment of 43 eyes with severe ocular surface disorders. All subjects underwent KLAL to achieve suitable conditions for consecutive PK. Due to failures of primary treatment in 17 eyes (39%), the KLAL was repeated. PK was performed in all the patients at 9-12 months after KLAL. As a retrospective study we analyzed data from the medical records including the preoperative and postoperative best corrected visual acuity, corneal clarity, surgical outcomes and complications, postoperative intraocular pressure, graft rejection, and other comorbidities and complications.

**Results:**

The preoperative visual acuity ranged from light perception to 0.01. The final improvement of visual acuity within a gain of one or more lines with the Snellen chart, including the results of successive surgical treatments after PK, was achieved in 23 operated eyes (53%). Early graft rejection was observed in 4 eyes (9%). In 3 eyes, it was manifested as endothelial rejection, and in 1 eye, as combined endothelial and epithelial rejection. PK failure requiring repetitive PK was present in 14 eyes (32%). Phthisis bulbi developed in 6 eyes (14%). Glaucoma or ocular hypertension was reported in 25 eyes (58%). A majority were treated with up to 3 topical agents or referred for trabeculectomy in 3 cases, transscleral cyclophotocoagulation in 2 eyes, and EX-PRESS glaucoma shunt implantation in 3 cases.

**Conclusions:**

Successful KLAL carries a high risk of subsequent PK failure. Visual function remains the second aim of treatment; the primary one is to stabilize the surface.

## 1. Introduction

Limbal stem cell deficiency (LSCD) is characterized by the reduction or loss of the stem cells in the limbus. These stem cells are vital for the repopulation of the corneal epithelium and the barrier function of the limbus. The corneal epithelial cells undergo constant renewal and regeneration. Cells from the surface are desquamated and replaced by proliferating basal epithelial cells from the periphery. The destruction or loss of these cells results in epithelial breakdown and persistent epithelial defects, corneal conjunctivalization, neovascularization, corneal scarring and chronic inflammation, thick fibrovascular pannus, ulceration, corneal melting, and consecutive perforation [1, 2]. It also contributes to the loss of corneal clarity, which is essential for good vision. Other problems are chronic or recurrent pain, redness, and photophobia.

LSCD may be primary in neurotrophic or endocrine keratopathy, aniridia, or erythrokeratoderma) or secondary to external factors, such as chemical or thermal burns, ionizing or ultraviolet radiation, Stevens-Johnson syndrome (SJS), ocular cicatricial pemphigoid, contact lens wear, microbial infections, tumors, or multiple ocular surgeries [1, 3–5].

The management of LSCD depends on whether the condition is unilateral or bilateral and involves some or all of the limbal stem cells [1, 6]. In cases of transient limbal injury, conservative medical treatment may sometimes be sufficient. However, larger or total limbal stem cell deficiency requires surgical management. The choice of treatment methods and the prognosis for successful surgery depend on many factors, [3] such as concomitant lid pathology, dry eye, and uncontrolled systemic disorders. The reconstruction of an appropriate limbal microenvironment involves limiting and controlling the inflammation, improving the tear film, and promoting the differentiation of the corneal epithelial cells with medical and surgical methods [1, 3, 6, 7].

From the first ocular surface transplantations performed by Thoft, that is, conjunctival transplantation in 1977 and keratoepithelioplasty in 1984, several surgical techniques have been developed to treat and to reconstruct severely damaged ocular surface epithelia [6]. They include autologous and allogenic limbal transplantation, simple limbal epithelial transplant (SLET), cultivated limbal epithelial transplantation (CLET), cultivated oral mucosal epithelial transplantation (COMET), amniotic membrane transplantation, keratolimbal allograft (KLAL), keratoprosthesis, and synthetic corneas. The options include living-related conjunctival limbal allografts (lr-CLAL), cadaveric KLAL, combined lr-CLAL and KLAL, and ex vivo expanded limbal allograft. For unilateral disease, conjunctival limbal autograft may be the best option. For bilateral disease, allogeneic tissues are needed as stem cell sources [3, 8–10].

The restoration of the ocular surface and final visual acuity improvement in patients with partial or total limbal stem cell deficiency often requires multistage and repetitive surgical treatments to regenerate the normal corneal epithelium and to create stable conditions for the final optical penetrating keratoplasty (PK). Reconstructive surgery involving simultaneous limbal transplantation and PK has poorer long-term outcomes. It carries a high risk of very serious complications and frequently requires successive intensive medical and surgical treatments [1, 11].

The aim of this study was to report on a sample of patients who underwent surgical treatment for severe corneal and ocular surface disorders. The surgical treatments, anatomical and functional results, and treatment complications in this group of patients are discussed.

## 2. Material and Methods

The study was a retrospective review of the surgical treatment of 43 eyes with severe ocular surface disorders. The operations were performed between January 1, 2010, and August 31, 2018, at the Ophthalmology Department of Saint Barbara Hospital, Trauma Center, Sosnowiec, Poland. The analyzed data from the medical records included demographics, medical histories, the preoperative and postoperative best spectacle-corrected visual acuity (BSCVA) measured in accordance with the Snellen visual acuity (VA) chart, corneal clarity, surgical outcomes and complications, postoperative intraocular pressure, graft rejection and other comorbidities and complications, and the results of the accessory examinations (microbial tests). Surgeries were performed by two experienced surgeons (KK, DD). All of the patients signed an informed consent form before undergoing any surgical procedures. Under Polish law, this retrospective observational study did not require the approval of a local bioethics committee.

The patients with severe bilateral ocular surface pathology with total LSCD usually required multistage surgical treatment. The clinically based total LSCD diagnosis was based on medical histories, patient observations, and treatment responses. The clinical manifestations of total LSCD were nonhealing persistent corneal epithelial defects, which often led to ulcerations, corneal melting or perforation, corneal superficial and deep neovascularization, fibrovascular pannus, scarring, keratinization and calcification of the cornea, severe limbal ischemia in four quadrants, and persistent limbal inflammation. The first step in the treatment of all of the patients who had been diagnosed with total LSCD and who had undergone complete ocular examination was 360° KLAL. The main purpose was the achievement of suitable conditions for consecutive optical PK. The indications for the procedure included chemical burns, thermal burns, and corneal postinflammatory and severe posttraumatic scars of the ocular surface.

In many cases, the reconstruction of the ocular surface required prior conjunctival fornix restoration, symblepharolysis, ankyloblepharolysis, and lid procedures.

The KLAL involved the preparation of the donor tissue, the 360° corneoscleral ring that remains after corneal trephination for PK, from cadaveric eyes. Oversized donor grafts with 2–3 mm scleral rims were preserved in a cold storage medium of Eusol-C solution (Alchimia, Srl, Ponte S. Nicolo, Italy). During surgery, the posterior one-half to two-thirds of the stromal and scleral tissues were removed by lamellar dissection with a sharp rounded steel crescent blade. The free-hand technique was used. A 360° conjunctival peritomy was performed by exposing the limbus. All of the corneal pannus and corneal irregularities were smoothed and removed with a rounded blade. Hemostasis was obtained by the topical application of 10% phenylephrine. The keratolimbal donor allograft was positioned and sutured with 7-0 vicryl sutures to the recipient scleral bed. The corneal part of the allograft usually fitted closely to the recipient cornea. Each recipient's primary pushed-back conjunctiva was sutured tightly with 10-0 vicryl 2 mm outside the limbus. The patients were hospitalized for 1–2 days after KLAL. They were followed up 2 weeks after hospitalization and monthly for 6 months.

In the patients in whom a stable ocular surface was obtained, optical PK was subsequently performed. Criteria for surgery continuation were as follows: no epithelial defects prior to PK, no conjunctival ingrowth on corneal central area, and no superficial neovascularization. For the PK, a Hanna vacuum trephine system (Moria Inc., Antony, France) was used. The graft diameter was 7.5–8.5 mm with an oversize of 0.5–0.75 mm. The donor-recipient junction was sewn with 10-0 nylon interrupted sutures. All of the patients were hospitalized for the first 2–5 days after PK. They were followed up every 2 weeks for 2 months, monthly for a minimum of 6 months and at various intervals thereafter.

Surgery was followed by the intensive management of epithelialization, local and systemic immunosuppression and control of inflammation, and the management of coexisting glaucoma and other comorbidities and complications.

All of the surgical procedures were performed under general anesthesia. The donor corneas originated from the authors' own or cooperative tissue banks.

XLSTAT-Biomed software (Addinsoft SARL, France) was used for statistical analysis, including the calculation of the means and standard deviations. The outcome variables were not assumed to have a normal distribution. Therefore, one-way analysis of variance was used to compare the baseline characteristics and postoperative outcomes regarding the causes of corneal disorders in the subgroups. A value of <0.05 was considered statistically significant.

## 3. Results

Thirty-five study subjects suffered from unilateral disease, while bilateral LSCD was present in five patients. In a study period 43 eyes of 40 patients with severe ocular surface disorders leading to LSCD received KLAL and consecutive PK at 9–12 months after the KLAL. This patient group consisted of 4 females, 4 eyes with the mean age at the time of KLAL being 52.25 ± 14.72 (range 37–71 years), and 36 males, 39 eyes with the mean age at the time of KLAL being 42.65 ± 12.29 (range 26–73 years). In 3 patients with LSCD after chemical burns in both eyes, KLAL was performed.

The primary causes of total LSCD that resulted from severe corneal disorders and required KLAL are presented in Table 1.

To create suitable conditions for PK in 17 eyes (39%), the KLAL was repeated. The anatomical and functional success of this treatment was defined as the absence of persistent epithelial defects and corneal conjunctivalization and neovascularization on the corneal edge of the graft.  Table 2 presents the characteristics of primary KLAL failure.

More than two KLAL procedures were not necessary in these subjects.

Because of the complex nature of the underlying pathologies, additional one-time or multiple surgical procedures were performed subsequently. To achieve final useful visual acuity and to manage the failure of the primary surgical interventions, the eyes treated for LSCD initially with KLAL and PK required successive surgical treatments.

The preoperative BSCVA on Snellen charts ranged from light perception to 0.01. The final improvement of visual acuity within a gain of one or more lines with the Snellen test, including the results of successive surgical treatments after PK, was achieved in 23 operated eyes (53%). Because of the coexisting ocular diseases, such as glaucoma or retinal disorders, the final visual outcome of the treatment was limited.  Table 3 presents the successive procedures that were performed to improve visual acuity and final BSCVA.

Despite repeated surgical treatments and intensive anti-inflammatory and immunosuppressive treatments, phthisis bulbi developed in 6 eyes (14%).

In 2 eyes, successive PK after KLAL was not necessary because of the remission of corneal neovascularization and the return of satisfactory corneal transparency and visual acuity. The phacoemulsification with PCIOL implantation was sufficient for improving final visual acuity. Those patients were not included in this review.

The post-KLAL medical treatment included a topical steroid (dexamethasone 7 times per day), broad-spectrum antibiotics (fluoroquinolones [moxifloxacin or levofloxacin]), aminoglycosides (gentamycin), and systemic immunosuppression, including steroids (methylprednisolone 4–16 mg BID, and tapered), cyclosporine A (100 mg, QD), azathioprine (50 mg BID), and mycophenolate mofetil (250–500 mg BID). Persistent intensive lubrication was very important for creating good conditions for epithelialization and maintaining the success of the surgical and medical treatments. Topical antimicrobial therapy was routinely applied for 21 days and extended if needed. The steroid doses were tapered (1 less drop each month) to a maintenance treatment of 5 times per day. The modifications were applied on the basis of the progress of the disease. Systemic long-term immunosuppression was continued with one of medications or in combination of cyclosporine A (100 mg, QD), azathioprine (50 mg BID), and mycophenolate mofetil (250–500 mg BID).

PK was performed in all the patients at 9–12 months after KLAL. In 7 eyes (16%), PK and cataract surgery with PCIOL implantation were performed.

Because of the high risk of rejection with PK after KLAL, topical and systemic steroid medications were continuously administered postoperatively. Intensive immunosuppression and anti-inflammatory and antibiotic therapies were also administered. In addition, the patients were treated with cycloplegic and antiglaucomatous medications, if necessary. All of the patients were followed up every 2 weeks for a period of 2 months, monthly for a minimum of 6 months and at varying intervals thereafter. The mean observation time was 24 months (1–60 months).

There were many complications from the two-step surgical transplantation treatment. The most common was persistent epithelial defect, observed in 27 eyes (63%), resulting from the impaired tear production and decreased corneal sensitivity that was observed in all groups of patients requiring surgical treatment. The treatment of the erosions consisted of intensive lubrication, the application of solution of 20% autologous serum combined with an antibiotic agent and dexamethasone. In 5 eyes, an amniotic membrane was also applied for 2 weeks to support reepithelialization.

Early graft rejection was observed in 4 eyes (9%). In 3 eyes, it was manifested as endothelial rejection, and in 1 eye, as combined endothelial and epithelial rejection. All 4 cases were successfully treated with intensive topical and systemic anti-inflammatory and immunosuppressive medications.

**Figure 1:**
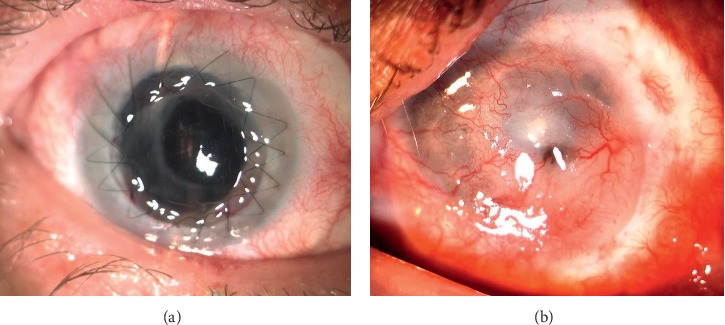
(a). Successful keratoplasty after KLAL with double running suture without vascular invasion crossing limbal border of the KLAL graft. (b) Total failure—superficial and deep vascular ingrowth into corneal bottom after PK with partial melting of both grafts.

PK failure requiring repetitive PK was present in 14 eyes (32%). Mean to time to graft failure was 8.2 ± 5.8 months. Nonmelting graft failure (23%) was attributed to stromal vascularization with partial ocular surface conjunctivalization (2 eyes), graft rejection with persistent edema (7 eyes), and scarification of the stroma (1 eye). Keratomalacia occurred in 4 eyes (9%). Neovascularization in the graft-host interface, as that in the midperiphery of the corneal graft, was caused by direct contact with the corneal limbus and immunological mechanisms in the transplanted tissues. Graft melting (keratomalacia) was observed mainly in the PK for postinflammatory viral reinfection. Intensive antiviral and anti-inflammatory medications were required.  Figure 1 presents ocular surface conditions in successful and failed cases.

Preoperative glaucoma recognized was present in 14 eyes (32%). All patients were treated with at least 1 antiglaucoma agent (timolol or brimonidine or brinzolamide or combination of them). Glaucoma or ocular hypertension in postoperative period, despite 1 application of PK peripheral iridectomy in 11 eyes (26%), was reported in 25 eyes (58%). A majority were treated with 1 or 3 topical agents (timolol, brimonidine, and brinzolamide) or referred for trabeculectomy in 3 cases, transscleral cyclophotocoagulation in 2 eyes, and EX-PRESS glaucoma shunt implantation in 3 cases. The main reason for glaucoma was coexisting preoperative disease, intensive steroid therapy, and shallow anterior chambers in the eyes after keratoplasty. Anatomical reasons urged us to surgical intervention, especially in the eyes of an unsatisfactory response to topical treatment.

## 4. Discussion

The limbal stem cell population is responsible for corneal epithelial renewal and repair, which are crucial for corneal transparency and good vision [2, 12]. LSCD, both primary and secondary, leads to epitheliopathy, persistent epithelial defects, inflammation of the corneal surface with consecutive conjunctivalization and neovascularization of the cornea, symblepharon and ankyloblepharon formation, shortening of the fornices, and goblet cell, mucin and aqueous tear deficiency [13–15].

Liang et al. [16] demonstrated the prevalence and threat of surface deficits and the augmentation of the long-term success of KLAL for eyes with total LSCD through the application of corrective measures. The maintenance of the reservoir of the corneal epithelial cells is necessary for limbal transplantation. The success of corneal transplants is attributed to a combination of anatomical, physiological, and immunological properties that prevent the induction and expression of potentially destructive immune responses to the transplanted tissue [17].

Simultaneous PK and limbal transplantation carry a high risk of failure. The two-step surgical approach, the first for reconstructing and sustaining ocular surface stability and the second for improving visual acuity, is preferable and strongly emphasized in the literature [12–16, 18].

It should be noted that safety and the reduction of potential postoperative complications also depend on the time of and the delay in the application of KLAL and optical PK. In the present study, the range was 9–12 months. Shimazaki et al. [19] proposed a delay of successive optical surgical procedures for at least several months after ocular surface reconstruction. The application of keratoplasty to inflamed and vascularized corneas increases the risk of immunologic rejection and epithelial problems. Solomon et al. [20] modified their surgical strategy and adopted a two-step approach by first performing KLAL and, subsequently, PK at 3–6 months.

The choice of treatment method, simultaneous or separated KLAL and PK, also depends on the availability of donor corneal tissue, as indicated by Han et al. [7]. However, they emphasized that the application of strong systemic and topical immunosuppression did not pose an increased risk of rejection in patients who underwent KLAL concurrently with PK in comparison to those who received KLAL only. However, Han et al. also recorded the concurrent rejection of the limbi and central grafted corneas in four eyes, two of which experienced irreversible graft failure.

The abovementioned success of one surgical procedure is in accordance with the results of Ilari and Daya [21] who found no difference in KLAL survival with the simultaneous or subsequent PK. However, KLAL combined with PK appeared to have a shorter survival time than KLAL followed by PK.

The survivability of the first KLAL in the patients in the present study was 60%. Solomon et al. [20] performed successive KLAL procedures after the failure of the first KLAL in 11 of 39 study group patients. Nine eyes required a second; 1 eye, a third; and 1 eye, a fourth KLAL. The prognosis for successful KLAL survival was probably related to persistent inflammation, severe dry eye, or the asymptomatic and progressive rejection of the KLAL. Thus, the survivability for KLAL, especially repeated applications, requires more intensive immunosuppression for a prolonged, if not indefinite, period [16].

Shi et al. [22] compared simultaneous KLAL and PK to another surgical technique: total PK for the treatment of severe corneal burns with total LSCD. They reported fewer complications and a better prognosis for the combined surgical approach. They stressed that the two-stage approach may act as a physical barrier against the invasion of the corneal stroma by the vessels and immune cells. In addition, corneoscleral transplantation is high-risk keratoplasty because of the oversized graft with a large number of antigens in the large donor grafts.

Systemic immunosuppression plays a very important role in the maintenance of clear and functional grafts. The rate of immune reactions is the result of the high immunogenic stimulus of the limbal transplant related to the presence of Langerhans cells and HLA-DR antigens, which play a crucial role in the afferent arm of the allograft rejection [23, 24].

The results of the application of systemic immunosuppressive medication after ocular surface reconstruction in severe LSCD are concordant with those of Holland and Schwartz [8]. The tapering of the two or three systemic immunosuppressants in concert rather than the discontinuation of one concurrent with the continuation of the other two at high doses is more desirable. It reduces the risk of side effects from the dangerous medications.

The increased intraocular pressure and secondary glaucoma observed in the present study despite the application of one PK peripheral iridectomy is more frequent than the result after procedures involving only the central part of the cornea. The current results are comparable to those obtained by Ti et al. [25], Ang et al. [26], and Jonas et al. [27] after the application of other tectonic procedures involving the peripheral cornea.

For the best final results of treatment, including the improvement of visual acuity, elective corneal and lens surgery, often multistage and repetitive, are necessary. In this study group, the most frequent procedure was cataract surgery, alone (20%) or combined with re-PK (7%), as well as re-PK because of allograft failure or rejection, corneal scarification, or neovascularization (29%).

The functional result of the primary treatment of total LSCD is not the main outcome. Surface reconstruction gives ability to improve vision offering the patient successful optical keratoplasty. PK in eyes with inactive primary disease and without inflammation leads to better and more predictive results.

In conclusion, because of the complex nature of the total LSCD, successful KLAL still carries a high risk of subsequent PK failure. Visual function remains the second aim of treatment. Further investigations and the development of new surgical techniques and appropriate immunosuppressive approaches are still necessary for improving the final results of treatment.

## Figures and Tables

**Table 1:** Causes of total limbal stem cell deficiency (% in brackets).

Cause of total LSCD	Total (*N* = 43) *N* (100%)	Female (*n* = 4) *n* (9.3%)	Male (*n* = 39) *n* (90.7%)
Chemical burn	25 (58.1)	2 (4.7)	23 (53.5)
Thermal burn	8 (18.6)	0	8 (18.6)
Postinflammatory scar	6 (13.9)	2 (4.7)	4 (9.3)
Posttraumatic scar	2 (4.7)	0	2 (4.7)
Stevens-Johnson syndrome	2 (4.7)	0	2 (4.7)

**Table 2:** Characteristics of primary KLAL failure.

Cause of KLAL failure	Total (*N* = 17) *N* (100%)	Chemical burn (*n* = 6)	Thermal burn (*n* = 1)	Postinflammatory scar (*n* = 6)	Posttraumatic scar (*n* = 0)	Stevens-Johnson syndrome (*n* = 2)
Primary failure	2 (11.8)	1 (16.7)				1 (50.0)
Rejection	2 (11.8)	1 (16.7)				
Persistent epithelial defect	3 (17.6)	1 (16.7)		2 (33.3)		
Corneal neovascularization	7 (41.2)	2 (33.3)	1 (100.0)	2 (33.3)		1 (50.0)
Conjunctivalization	3 (17.6)	1 (16.7)		2 (33.3)		

**Table 3:** Successive surgical treatments after penetrating keratoplasty and final best spectacle-corrected visual acuity.

Surgical technique	Total (*n* = 35) *N* (%)	Final BSCVA (range)
Re-PK	10 (28.6)	HM – 0.2
Re-PK with cataract surgery and PCIOL implantation	3 (8.6)	0.1–0.3
Cataract surgery with PCIOL implantation	7 (20.0)	0.1–0.5
Secondary PCIOL implantation	2 (5.7)	0.02–0.3
Transscleral fixation of IOL	3 (8.6)	0.05–0.1
Glaucoma surgery	8 (22.6)	LP – 0.01
Pars plana vitrectomy (PPV)	2 (5.7)	HM – 0.1

CF: counting fingers; HM: hand movements.

## Data Availability

The patients' data used to support the findings of this study are included within the article.
